# Attachment styles and defense mechanisms as predictors of marital satisfaction in Iranian couples

**DOI:** 10.3389/fpsyg.2026.1659840

**Published:** 2026-03-19

**Authors:** Fatemeh Khodabakhsh Nejad, Mahdi Mahmoudi, Michaela Hiebler, Human-Friedrich Unterrainer

**Affiliations:** 1 Faculty of Psychotherapy Science, Sigmund Freud University, Vienna, Austria; 2 Faculty of Medicine, Sigmund Freud University, Vienna, Austria; 3 Addiction Research Hub (A-R-H), Grüner Kreis Ltd., Vienna, Austria; 4 Department of Religious Studies, University of Vienna, Vienna, Austria; 5 Division of Psychiatry and Psychotherapeutic Medicine, Medical University of Graz, Graz, Austria

**Keywords:** attachment styles, defense mechanisms, Iranian couples, marital satisfaction, relationship dynamics

## Abstract

**Background:**

Although prior research has explored the roles of attachment styles and defense mechanisms in marital satisfaction, limited attention has been given to how these two factors interact. This study investigates the combined influence of attachment styles and defense mechanisms on marital satisfaction in Iranian couples.

**Methods:**

A total of 228 participants (114 couples) completed three online questionnaires: The Revised Adult Attachment Scale, the Marital Intimacy Needs Questionnaire, and the Defense Style Questionnaire. Hierarchical multiple regression with interaction terms was used to examine the moderating effects of attachment styles and defense mechanisms on marital satisfaction.

**Results:**

Analyses showed several connections between marital satisfaction and attachment styles as well as defense mechanisms. Notably, in the prediction of marital satisfaction, secure attachment enhanced marital satisfaction when paired with mature defense mechanisms.

**Discussion:**

Our findings highlight potential interactive effects of attachment styles and defense mechanisms in shaping marital satisfaction. The results emphasize the need for therapeutic interventions that integrate both attachment-based and defense-oriented approaches to enhance marital well-being.

## 1 Introduction

### 1.1 Attachment styles and marital satisfaction

According to attachment theory, proposed by John Bowlby (1980), humans are biologically driven to seek closeness from significant others to gain emotional security. Therefore, attachment refers to the emotional bond formed with those who can meet basic needs (Simpson et al., 2021). This system is activated by perceived threats, prompting proximity-seeking for reassurance (Bowlby, 1988). As an evolutionarily rooted psychobiological system, attachment supports individual survival and social cohesion (Lahousen et al., 2019). Without social connection, humans may struggle not only to reproduce but also to meet basic survival needs (Carter, 2014). These early patterns lay the foundation for later emotional, cognitive, and social development and predict future relational dynamics (Lahousen et al., 2019).

Marital satisfaction refers to the extent to which individuals feel their needs and expectations are fulfilled in a relationship (Gelles, 1995). Partners who are unresponsive or emotionally distant may contribute to dissatisfaction (Kunce and Shaver, 1994). Such patterns – marked by emotional neglect or lack of support – are linked to declining marital satisfaction (Simpson et al., 2021). Recent perspectives regard marital satisfaction as a *multidimensional and dynamic construct* that includes emotional, cognitive, and behavioral dimensions of relational well-being, evolving over time (Dobrowolska et al., 2020). Ongoing dissatisfaction can lead to emotional or legal separation, with significant psychological and social consequences (Leopold, 2018).

To understand the roots of marital satisfaction, researchers have focused on attachment styles. Attachment theory offers a useful framework for analyzing romantic relationships (Mikulincer and Shaver, 2016), as attachment patterns, mostly formed in early childhood, significantly shape adult romantic bonds (Hazan and Shaver, 1987). Though romantic attachment differs from early attachment through reciprocity and sexual interaction, it still operates as an attachment system (Sadock et al., 2017). Adult attachment styles are generally classified as secure or insecure, with the latter including anxious, avoidant, and fearful-avoidant (disorganized) subtypes (Fitzpatrick and Lafontaine, 2017; Williams and Riskind, 2004). Studies consistently show that secure attachment is positively linked to marital satisfaction, while insecure attachment predicts lower satisfaction (Amani et al., 2024; Besharat et al., 2014; Bühler et al., 2021; Ghiasi et al., 2024; Martins et al., 2023).

Commitment is a fundamental element of interpersonal relationships, including marriage, and serves as a cornerstone of social life (Michael et al., 2016). Insecure attachment styles are often linked to lower levels of commitment, which can increase the risk of infidelity and reduce marital satisfaction (Simpson, 1990). Freeman et al. (2023) reported that people with higher anxious attachment levels typically tend to initiate relationships with lower levels of commitment. According to Stanley et al. (2010), people exhibiting avoidant attachment are likely to avoid increasing commitment due to their discomfort with closeness and intimacy.

Correspondingly, trust is another key factor in marital satisfaction. Couples who trust each other are more likely to share thoughts, emotions, and needs openly, without fear of judgment or rejection (Feeney, 2016). In contrast, insecurely attached individuals often struggle with trust, leading to dissatisfaction in the relationship (Simpson, 1990). Research shows that secure attachment is associated with higher levels of trust, commitment, satisfaction, and mutual support (Simpson, 1990). Avoidantly attached individuals often use a strategy known as deactivation, emotionally distancing themselves and limiting interaction with their partners (Shaver and Mikulincer, 2006). They also tend to avoid conflict and are less likely to seek or offer support during emotionally intense moments (Li and Chan, 2012).

Anxious attachment is linked to lower marital satisfaction due to heightened fears of abandonment and rejection (Mendez, 2023). Individuals with this style often seek excessive closeness to feel secure, but these efforts - known as hyperactivation strategies–can intensify emotional demands and conflict (Mikulincer and Shaver, 2012; Simpson and Rholes, 2017). Their fear of rejection may lead them to doubt their partner’s support, viewing it as insincere or insufficient (Collins and Feeney, 2004). In trying to gain reassurance, they may devalue themselves, over-focus on their partner’s needs, and adopt a caregiving role at the expense of their own well-being (Mikulincer and Shaver, 2012). These self-neglecting behaviors, rooted in insecurity, often increase relational stress and undermine emotional intimacy, further reducing marital satisfaction. Securely attached individuals generally have a positive self-view, low fear of rejection, and can form emotionally close relationships with ease (González-Ortega et al., 2020). They see their partners as reliable and responsive and manage conflict constructively (Bowlby, 1980). They are more likely to seek and receive support (Sivandian and Besharat, 2019) and view conflict as a chance to improve mutual understanding rather than a threat (Pietromonaco et al., 2004). Lastly, research highlights that attachment insecurity is expressed through distinct emotion-regulation strategies, with anxious individuals typically showing *hyper-activation* and avoidant individuals showing *de-activation* tendencies, both shaping relational dynamics and satisfaction (Girme et al., 2021).

### 1.2 The role of intimacy in marital satisfaction

As stated by Bowlby (1988) “attachment theory regards the propensity to make intimate emotional bonds to particular individuals as a basic component” (p. 119). Intimacy lies at the heart of many romantic relationships (Sternberg and Grajek, 1984) and is widely recognized as essential for marital satisfaction (Yang and Sari, 2025). Bagarozzi (1997), whose Marital Intimacy Needs Questionnaire was utilized in this study, described intimacy as a dynamic, reciprocal, and interactive process between spouses. Intimate relationships offer a unique sense of emotional security that is not easily replicated in other forms of connection (Bradbury and Bodenmann, 2020). Adults who experience fear of intimacy often avoid social interactions, prefer solitude, and may even display interpersonal hostility, viewing intimacy as a threat to their personal identity (Schultz and Schultz, 2016). Research has shown that adults who experience fear of intimacy typically struggle with self-disclosure and, consequently, have difficulty forming and maintaining close relationships, and they often report lower levels of marital satisfaction (Besharat et al., 2014).

Individuals with an avoidant attachment style tend to avoid emotional closeness and self-disclosure in romantic relationships (Mikulincer and Shaver, 2012). Unlike securely attached individuals, they are less likely to seek comfort from their partners and often struggle with connection and intimacy (González-Ortega et al., 2020). Emphasizing independence and self-sufficiency, avoidant individuals may devalue intimacy and feel little need for close bonds (Sivandian and Besharat, 2019). Their emotional distancing and reluctance to commit can increase the risk of infidelity, further undermining marital stability (Mikulincer and Shaver, 2012). Since many marital issues stem from concerns about availability and responsiveness (Kobak et al., 2016), avoidant behaviors may intensify these insecurities. Although avoidant individuals often fear intimacy, research shows that both avoidant and anxious attachment styles are linked to low expectations of acceptance, contributing to fear of intimacy through different strategies (Finzi-Dottan and Abadi, 2024). In contrast, securely attached individuals – low in both anxiety and avoidance - tend to feel self-worthy, trust others, and are comfortable with emotional closeness (Collins and Feeney, 2004). They openly express emotions, view relationships positively, and experience greater intimacy and mutual dependence with their partners (Simpson and Rholes, 2017; Sivandian and Besharat, 2019).

### 1.3 Defense mechanisms and marital satisfaction

According to Anna Freud (1936), defense mechanisms are unconscious strategies used by the ego to protect individuals from anxiety and unacceptable internal impulses. These mechanisms help buffer the negative impact of insecure attachment styles on mental health (Laczkovics et al., 2020). For example, avoidantly attached individuals often rely on deactivation strategies - denying attachment needs - which can lead to unstructured expressions of anger or hostility (Mikulincer and Shaver, 2016). Andrews et al. (1993) grouped defense mechanisms into three types: mature (e.g., sublimation, humor), neurotic (e.g., pseudo-altruism, reaction formation), and immature (e.g., denial, passive aggression). Immature defense mechanisms, which involve greater distortion of reality, are viewed as the least adaptive (Perry and Bond, 2017). Clinically, they are often linked to depression and maladaptive behaviors (Sivandian and Besharat, 2019). Individuals with lower psychological adjustment are more likely to use immature defense mechanisms (Nam et al., 2019), which have been associated with lower marital adjustment, greater conflict, and reduced satisfaction (Ungerer et al., 1997). Neurotic defense mechanisms, though less extreme, allow individuals to keep distressing thoughts unconscious while maintaining a mostly accurate view of reality (Békés et al., 2021).

Mature defense mechanisms reflect an individual’s advanced ability to cope with reality and handle social and emotional challenges without distorting the external world (Ma et al., 2024). Unlike neurotic and immature defense mechanisms, mature defense mechanisms reduce anxiety while maintaining an accurate perception of reality (Békés et al., 2021). Strategies like humor and sublimation help individuals become more aware of their emotions, communicate openly with spouses, accept support, and solve problems effectively (Sivandian and Besharat, 2019). Research shows couples using mature defense mechanisms experience greater marital adjustment than those relying on immature defense mechanisms. Additionally, mature and immature defense mechanisms significantly moderate the link between secure attachment and marital adjustment, highlighting their crucial role in romantic relationships (Sivandian and Besharat, 2019). Despite extensive research linking attachment styles to marital satisfaction, few studies have examined how defense mechanisms interact with attachment orientations to influence relationship quality - particularly in non-Western cultural contexts such as Iran. Addressing this gap, the present study explores the combined role of attachment and defense processes in explaining variations in marital satisfaction.

### 1.4 Study objectives and hypotheses

The present study aimed to examine the relationships among attachment styles, defense mechanisms, and marital satisfaction in Iranian couples. Specifically, we sought to (a) assess how attachment styles and defense mechanisms predict marital satisfaction, and (b) explore whether defense mechanisms moderate the relationship between attachment and marital satisfaction.

Hypotheses:

1. Insecure attachment (anxious and avoidant) will be negatively associated, and secure attachment positively associated, with marital satisfaction.
2. Mature defense mechanisms will be positively associated, and immature defense mechanisms negatively associated, with marital satisfaction.
3. Defense mechanisms will moderate the relationship between attachment and marital satisfaction.

## 2 Materials and methods

### 2.1 Participants and procedure

The study collected online data from 114 couples (228 individuals) using convenience sampling. Participation was voluntary, informed consent was obtained, and pseudonyms were used to ensure confidentiality. Ethical approval was granted by the Ethics Committee of Sigmund Freud University Vienna, Austria.

Participants were married heterosexual couples recruited through online advertisements, counseling centers, and social media platforms in Tehran. Interested individuals received a study link directing them to an online consent form and questionnaires. To encourage honest responses, participants were offered a free consultation. To be included, participants had to be married for at least one year and living with their spouse at the time of the study. All participants provided informed consent and completed the same set of questionnaires individually and anonymously. Responses from 295 participants were initially collected; 67 were excluded due to incomplete or unmatched partner data, resulting in a final sample of 228 participants.

### 2.2 Psychometric assessment

This study employed three standardized psychometric instruments in Farsi (Persian) language. All Persian versions had been previously validated in earlier research.

#### 2.2.1 Revised adult attachment scale–18 (RAAS-18)

Originally developed by Collins (1996) and adapted by Teixeira et al. (2019), this scale assesses three adult attachment styles - secure, anxious, and avoidant - across 18 items rated on a 5-point Likert scale (1 = Not at all characteristic of me; 5 = Very characteristic of me). Example items include “I find it easy to depend on others” (secure), “I worry about being abandoned” (anxious), and “I prefer not to show others how I feel deep down” (avoidant). In the present sample, internal consistency Cronbach *α* was found to 0.709 for secure, 0.757 for anxious, and 0.485 for avoidant attachment.

#### 2.2.2 Marital intimacy needs questionnaire-41 (MINQ-41)

Developed by Bagarozzi (2001), this 41-item instrument measures marital intimacy needs on a 10-point Likert scale (1 = Not at all important; 10 = Extremely important). Example items include “How important is it for you to share your deepest thoughts and feelings with your spouse?” and “How important is it for you to have physical intimacy with your spouse?” Reliability analysis revealed excellent internal consistency for the total scale (α = 0.944) and satisfactory values for the subscales addressing different types of intimacy: Emotional (α = 0.693), Psychological (α = 0.756), Intellectual (α = 0.807), Physical (α = 0.836), Sexual (α = 0.773), Spiritual (α = 0.828), and Aesthetic (α = 0.754).

#### 2.2.3 Defense style questionnaire-40 (DSQ-40)

Created by Andrews et al. (1993), this 40-item measure evaluates 20 defense mechanisms grouped into three defense styles–mature, neurotic, and immature–using a 9-point Likert scale (1 = Strongly disagree; 9 = Strongly agree). Example items are “I use humor to make difficult situations more bearable” (mature), “I often do things for others that I do not really want to do” (neurotic), and “I tend to blame others for my problems” (immature). The total scale demonstrated highly satisfying reliability (α = 0.844), with subscale reliabilities of α = 0.592 for Mature, α = 0.814 for Neurotic, and α = 0.648 for Immature defense styles.

### 2.3 Statistical analyses

Statistical analyses were conducted using SPSS version 27. Analyses included descriptive statistics, independent samples t-tests for gender differences, Pearson correlation coefficients to examine continuous relationships between variables and hierarchical multiple regression analysis to examine the unique and combined effects of attachment styles and defense mechanisms on marital satisfaction, including interaction effects.

## 3 Results

### 3.1 Descriptive statistics and gender differences

The sample comprised 228 participants (50% women, 50% men). Women (*M* = 39.64; range: 20–67 years of age) were significantly younger than men (*M* = 41.86, range: 21–71 years of age) (*p* < 0.05). Descriptive statistics for study variables, stratified by gender, are presented in Table 1. Women (*M* = 7.19) reported higher levels of anxious attachment compared to men (*M* = 5.12) (*p* < 0.01; Cohen’s *d* = −0.437), whereas men (*M* = 5.69) demonstrated higher levels of mature defense mechanisms than women (*M* = 5.24) (*p* < 0.05; Cohen’s *d* = −0.328). No significant gender differences were observed for marital satisfaction, secure attachment, avoidant attachment, neurotic defense mechanisms, or immature defense mechanisms.

**Table 1:** Gender differences in marital satisfaction, attachment styles, and defense mechanisms.

Variable	Women		Men		*t*-value	*d*
	Mean	SD	Mean	SD		
Marital satisfaction	506.77	182.45	506.17	184.01	−0.02	
Secure attachment	16.19	4.79	16.61	4.71	0.67	
Anxious attachment	7.19	5.57	5.12	3.72	−3.30**	−0.437
Avoidant attachment	13.63	4.34	12.95	3.69	−1.28	
Mature defense mechanisms	5.24	1.31	5.69	1.32	2.55*	0.338
Neurotic defense mechanisms	5.22	1.43	4.99	1.48	−1.21	
Immature defense mechanisms	4.44	1.16	4.46	1.19	0.11	

*p < 0.05; **p < 0.01; *N* = 228 (114 women, 114 men). Cohen’s *d* for significant gender differences.

### 3.2 Bivariate correlations

As displayed in Table 2, higher levels of marital satisfaction were related to higher levels of secure attachment (*r* = 0.148, *p* < 0.05) and lower levels of anxious attachment (*r* = −0.194, *p* < 0.01), avoidant attachment (*r* = −0.198, *p* < 0.01), and immature defense mechanisms (*r* = −0.220, *p* < 0.001). Analysis of the relationship between attachment styles and defense mechanisms revealed that more secure attachment was related to more mature (*r* = 0.157, *p* < 0.05) and neurotic (*r* = 0.149, *p* < 0.05) but less immature (*r* = −0.133, *p* < 0.05) defense mechanisms. More anxious attachment was related to more neurotic (*r* = 0.241, *p* < 0.001) and immature (*r* = −0.427, *p* < 0.001) defense mechanisms while avoidant attachment was only related to more immature defense mechanisms (*r* = −0.309, *p* < 0.001).

**Table 2:** Correlations between marital satisfaction, attachment styles, and defense mechanisms.

Variable	1	2	3	4	5	6	7
1. Marital satisfaction	1.000						
2. Secure attachment	0.148*	1.000					
3. Anxious attachment	−0.194**	−0.329***	1.000				
4. Avoidant attachment	−0.198**	−0.382***	0.420***	1.000			
5. Mature defense mechanisms	0.113†	0.157*	−0.075	0.021	1.000		
6. Neurotic defense mechanisms	0.007	0.149*	0.241***	−0.030	0.289***	1.000	
7. Immature defense mechanisms	−0.220***	−0.133*	0.427***	0.309***	0.284***	0.521***	1.000

†*p* < 0.10, *p < 0.05, **p < 0.01, ***p < 0.001; *N* = 228.

### 3.3 Hierarchical regression analysis

To examine the unique and combined effects of attachment styles and defense mechanisms on marital satisfaction, we conducted a hierarchical multiple regression analysis. This analytical approach was chosen because it allows us to: (1) control for potential confounding variables, (2) assess the incremental contribution of each predictor set, and (3) test interaction effects while maintaining statistical power.

The regression analysis proceeded in four steps (Table 3). Model 1 (demographics) explained 1.4% of the variance (adj*R*^2^ = 0.014, *F* = 2.59, *p* < 0.10), with age emerging as a significant negative predictor (*β* = −2.79, *p* < 0.05), while gender was not significant (*β* = −8.39, *p* = 0.366). Adding attachment styles in Model 2 explained 6.4% of the variance (adj*R*^2^ = 0.064, Δ*R*^2^ = 0.062, *F* = 4.09, *p* = 0.001); anxious attachment emerged as a marginal negative predictor (*β* = −5.25, *p* < 0.10). Model 3, which included defense mechanisms, explained 10.8% of the variance (adj*R*^2^ = 0.108, Δ*R*^2^ = 0.055, *F* = 4.43, *p* < 0.001), with mature defense mechanisms emerging as a significant positive predictor (*β* = 21.53, *p* < 0.05) and immature defense mechanisms as a significant negative predictor (*β* = −43.08, *p* < 0.01).

**Table 3:** Hierarchical regression analysis predicting marital satisfaction.

Predictor	Model 1	Model 2	Model 3	Model 4
Demographics				
Age	−2.79*	−2.98*	−3.05*	−3.10*
Gender	−8.39	17.36	16.78	3.83
Attachment styles				
Secure		2.50	1.13	20.16†
Anxious		−5.25†	−2.16	−3.79
Avoidant		−5.53	−3.92	−9.60
Defense mechanisms				
Mature			21.53*	86.24*
Neurotic			11.54	16.32
Immature			−43.08**	−59.33†
Interactions				
Secure × Mature				−3.73†
Anxious × Neurotic				0.22
Avoidant × Immature				1.31
Model statistics				
*R* ^2^	0.022	0.084	0.139	0.155
Adjusted *R*^2^	0.014	0.064	0.108	0.112
Δ*R*^2^	-	0.062	0.055	0.016
*F*	2.59†	4.09**	4.43***	3.59***

†*p* < 0.10, *p < 0.05, **p < 0.01, ***p < 0.001; *N* = 228. Values shown are unstandardized regression coefficients. All VIF values < 2.0, indicating no problematic multicollinearity.

The final model (Model 4), incorporating interaction terms, accounted for 11.2% of the variance in marital satisfaction (adj*R*^2^ = 0.112, Δ*R*^2^ = 0.016, *F* = 3.59, *p* < 0.001). Significant predictors included age (*β* = −3.10, *p* < 0.05) and mature defense mechanisms (*β* = 86.24, *p* < 0.05). Secure attachment (*β* = 20.16, *p* < 0.10), immature defense (*β* = −59.33, *p* < 0.10) mechanisms and the interaction between secure attachment × mature defense mechanisms (*β* = −3.73, *p* < 0.10) were only marginally significant. The marginally significant interaction between secure attachment and mature defense mechanisms might indicate that the relationship between secure attachment and marital satisfaction varies as a function of mature defense mechanism use.

Multicollinearity diagnostics were conducted for all predictors, with Variance Inflation Factor (VIF) values all below 2.0, indicating no concerning levels of multicollinearity among the predictor variables. Further analysis of the interaction effects through simple slopes analysis revealed distinct patterns. For the secure attachment × mature defense mechanisms interaction, the positive effect of mature defense mechanisms on marital satisfaction was strongest at low levels of secure attachment (*β* = 42.70, *p* < 0.01), moderate at medium levels (*β* = 25.01, *p* < 0.05), and non-significant at high levels (*β* = 7.31, *p* = 0.552). This pattern suggests that mature defense mechanisms are particularly beneficial for marital satisfaction among individuals with lower levels of secure attachment, with the benefit diminishing as secure attachment increases.

## 4 Discussion

Building on previous findings (Besharat et al., 2014; Mikulincer and Shaver, 2016), this study examined the role of attachment style in marital satisfaction. In addition, this research gives further insights in the combined effects of attachment styles and defense mechanisms on marital satisfaction (Sivandian and Besharat, 2019). In line with previous research, the findings indicate that women display higher levels of anxious attachment compared to men (Del Giudice, 2011, 2019). Furthermore, men appear to be more likely to adopt mature defense mechanisms. However, these findings contradict Abid and Riaz’s (2017) research, which found no significant gender differences in defense mechanism usage. This discrepancy may be due to variations in measurement tools, sample characteristics, or cultural influences. In line with our hypothesis, results show that (1) insecure attachment (anxious and avoidant) is negatively associated with marital satisfaction, that (2) mature defense mechanisms are positively associated, and immature defense mechanisms negatively associated, with marital satisfaction and that (3) defense mechanisms appear to moderate the relationship between attachment and marital satisfaction.

In detail, correlational analyses demonstrated a positive connection between secure attachment and marital satisfaction, while anxious and avoidant attachment styles – as well as immature defense mechanisms – appear to be linked to lower marital satisfaction. Notably, mature defense mechanisms showed only a marginal positive correlation with marital satisfaction (*r* = 0.113, *p* = 0.087). Regression and interaction analyses indicate that attachment styles and defense mechanisms together might explain a portion of the variance in marital satisfaction. In detail, mature defense mechanisms emerged as a relevant positive predictor of marital satisfaction with secure attachment emerging as an additional, but only marginally significant predictor. These results are consistent with the findings of Parooi et al. (2018), which highlighted the importance of early-formed attachment styles and defense mechanisms in shaping marital satisfaction (see Sivandian and Besharat, 2019 for further discussion).

Furthermore, the findings suggest that defense mechanisms may interact in their predictive effect on marital satisfaction. Secure attachment appears to be most advantageous when paired with mature defense mechanisms, as indicated by a marginally significant interaction effect (*β* = −3.73, *p* = 0.069). Interestingly, Sivandian and Besharat (2019) demonstrated that both mature and immature defense mechanisms moderate the association between secure attachment styles and marital adjustment. Their findings suggest that attachment styles and defense mechanisms are key predictors of marital adjustment. Additionally, previous studies have found a significant positive correlation between marital adjustment and mature defense mechanisms, whereas immature defense mechanisms show a significant negative correlation with marital adjustment (Navid et al., 2023). Additionally, age emerged as a significant negative predictor, potentially reflecting factors such as relationship fatigue, unresolved conflicts, shifting needs, or declining health over time (Korporaal et al., 2013).

### 4.1 Clinical implications

The findings provide valuable insights for clinicians, particularly those working in couples therapy or premarital counseling. By integrating attachment theory and defense mechanism profiles into assessment and treatment planning, therapists may be better able to address the underlying dynamics contributing to marital distress (Parooi et al., 2018). In particular promoting secure attachment patterns and the use of mature defense mechanisms may enhance relationship functioning and long-term satisfaction (Sivandian and Besharat, 2019).

### 4.2 Limitations

This study has several limitations. The sample consisted exclusively of Iranian couples, limiting the generalizability of findings to other cultural contexts. Additionally, the data relied on self-report measures rather than structured clinical interviews, raising concerns about subjective bias, interpretation, and response accuracy. The cross-sectional design further restricts causal inference. Future longitudinal research is necessary to explore developmental trajectories in attachment, marital satisfaction, and defense mechanisms over time. Moreover, incorporating contextual variables – such as cultural norms, socioeconomic status, and external stressors – could provide a more comprehensive understanding of the factors influencing marital satisfaction. As analyses were performed at the individual level, we could not account for dyadic interdependence. Future work should apply dyadic models such as the Actor-Partner Interdependence Model to better capture mutual influences within couples (Kenny et al., 2020; Bolger and Laurenceau, 2021).

### 4.3 Future directions

This study suggests that attachment styles and defense mechanisms as well as their interactions need to be considered in the prediction of marital satisfaction, especially secure attachment and mature defense mechanisms. More research will be needed to better understand how attachment styles and defense mechanisms - as well as potential further factors such as emotion regulation strategies - contribute to marital satisfaction in different relationship settings. Overall, this study underscores the importance of considering both attachment styles and defense mechanisms to understand marital satisfaction within cultural contexts.

## Data Availability

The raw data supporting the conclusions of this article will be made available by the authors, without undue reservation.
